# Sex differences in the relationship between hearing status and cognitive performance in individuals with mild cognitive impairment

**DOI:** 10.1002/alz70857_098695

**Published:** 2025-12-24

**Authors:** Nicole Grant, Natalie A. Phillips

**Affiliations:** ^1^ Concordia University, Montreal, QC, Canada; ^2^ Concordia University, Montréal, QC, Canada

## Abstract

**Background:**

Age‐related hearing loss (HL) is associated with poorer cognition and increased risk of developing dementia. However, few studies have explored if sex affects the association between HL and cognition. We investigated the relationship between two hearing measures and performance on cognitive screening and neuropsychological tests in males and females with mild cognitive impairment (MCI).

**Method:**

Participants included 260 older adults with MCI from the COMPASS‐ND study of the Canadian Consortium in Neurodegeneration in Aging. Participants were recruited from memory clinics and included normal hearing males (*n* = 93, mean age=70.9, mean education = 15.0, ApoE4 carrier=39%), HL males (*n* = 68, mean age=74.6, mean education = 15.3, %ApoE4 carrier=28%), normal hearing females (*n* = 63, mean age=70.3, mean education = 15.3, %ApoE4 carrier=38%), and HL females (*n* = 36, mean age=74.6, %ApoE4 carrier=42%). Groups did not differ in education, ApoE4 carrier status, or cardiovascular risk factors. Participants were categorized as normal hearing or HL based on a pure‐tone audiometry screening (normal hearing=pure‐tone threshold <25 dB HL at 2000 Hz). The Canadian Digit Triplet Test was used to measure speech‐in‐noise perception. Within each sex, we investigated if HL or speech‐in‐noise perception was associated with performance on the Montreal Cognitive Assessment (MoCA), Rey Auditory Verbal Learning (RAVLT), Brief Visuospatial Memory Test‐Revised (BVMT‐R), and a facename association test.

**Result:**

Females with HL were more likely to fail the MoCA than normal hearing females (X^2^ =  5.2, *p* = 0.02). This difference was not observed in males and it was not observed in females when hearing dependent items on the MoCA were excluded. No effects of hearing status were observed on the more comprehensive RAVLT, BVMT‐R, or Face Name Association test. Partial correlations (controlling for age) showed that speech‐in‐noise perception was significantly associated (*p* < .05) with performance on the MoCA (*r* = ‐0.17), RAVLT Immediate Recall (*r* = ‐0.19), and BVMT‐R Immediate Recall (*r* = ‐0.2) in males, but not females.

**Conclusion:**

These findings indicate differing associations between sex, HL, and cognitive performance, with implications for cognitive assessment. The findings suggest that the presence of MCI may be over‐estimated in females with HL when a simple screening test is used and highlight the importance of using comprehensive measures to assess cognition in older adults, especially those with HL.

